# Risk factors for fasting blood glucose control in middle-aged and elderly type 2 diabetes patients

**DOI:** 10.1097/MD.0000000000039322

**Published:** 2024-08-16

**Authors:** Nan-yue Kuang, Ye Hong, Jie-ping Chen, Hui Li, Na Tang

**Affiliations:** a Hospital of Xinjiang Production and Construction Corps, Urumqi, China; b Hospital of Xinjiang Production and Construction Corps, Urumqi, China.

**Keywords:** blood glucose control, medication use, risk factors, type 2 diabetes

## Abstract

This study aimed to investigate and analyze the medication use, fasting blood glucose control, and associated risk factors among residents with type 2 diabetes at the grassroots level in Xinjiang Production and Construction Corps. A multi-stage cluster sampling method was employed to conduct a questionnaire survey among residents aged 45 and above in battalions (communities) as the smallest unit. The medication use was recorded, and fasting blood glucose control was considered as the dependent variable. Logistic regression analysis was performed to identify the risk factors influencing fasting blood glucose control among different population characteristics. A total of 2316 participants were included in the study, of which 1072 were male (45.12%), 1418 were aged 65 and above (61.23%), 2031 were Han Chinese (87.69%), and 1551 were from the surrounding areas of Urumqi (66.97%). The main medications used among the top three classes were metformin, insulin, and α-glucosidase inhibitors. The treatment rate for type 2 diabetes was 71.80%, and the fasting blood glucose control rate was 27.98%. Multivariate analysis identified living outside the Urumqi surrounding area, age 65 and above, body mass index ≥ 24, abnormal blood lipids, and untreated hypertension as independent risk factors for poor fasting blood glucose control, while treatment was a protective factor for achieving blood glucose control. The treatment rate and fasting blood glucose control rate among grassroots residents with type 2 diabetes in Xinjiang Production and Construction Corps need improvement. Efforts should be made to enhance patient medication adherence and health management awareness through education. Targeted interventions should be implemented for high-risk populations with identified risk factors to reduce or delay the occurrence of diabetes and its complications, ultimately aiming to reduce mortality rates and improve quality of life.

## 1. Introduction

Type 2 diabetes mellitus (T2DM) has emerged as the third most prevalent cause of mortality globally, presenting a grave threat to public health.^[1,2]^ China, home to the largest population of individuals afflicted with diabetes, is witnessing a rapid increase in the prevalence of T2DM.^[3,4]^ The implementation of a nutritious diet, enhancements in lifestyle choices, weight regulation, pharmaceutical interventions, and management of other risk factors constitute pivotal facets of the regulation of diabetes progression.^[5]^ Moreover, these measures have proven efficacious in mitigating complications, enhancing the quality of life, and reducing medical expenditures. The Xinjiang Production and Construction Corps (hereafter referred to as “the Corps”) is situated in fragmented regions along the Xinjiang border and lacks comprehensive epidemiological data on patients with T2DM. This study is part of a series of research reports that focusing on the screening and management of common chronic diseases within the corps. The objective of this study was to ascertain the utilization of medications and the current status of fasting blood glucose (FBG) control among T2DM patients within the grassroots population of the Corps. Furthermore, we aimed to analyze the independent risk factors that influence FBG control, thereby establishing a foundation for targeted interventions within the Corps.

## 2. Data and methods

### 2.1. Survey objective

In the first stage, Urumqi, the capital of Xinjiang, was chosen as the study location, because it is a developed city with a strong economy. The research subjects were selected from the 104th Regiment of the 12th Division of the Xinjiang Production and Construction Corps in the Urumqi surrounding area, as well as a farm in the 4th Division of the Xinjiang Production and Construction Corps outside the Urumqi surrounding area. The inclusion criteria were as follows: Individuals aged ≥ 45 years who were permanent residents (residing for more than 6 months) and voluntarily agreed to participate in the study. Participants who met any of the following criteria: patients diagnosed with T2DM by a medical institution at the county level or above; T2DM diagnosis based on the “Primary Care Guidelines for Type 2 Diabetes” (2019 edition), excluding T1DM, other types of diabetes, pregnancy, lactation, and severe alcoholism; and regular use of ≥1 type of medication for T2DM treatment.

In the second stage, 2 separate samplings were conducted. In 2019, 1662 permanent residents from a specific company in the 12th Division of the Urumqi surrounding area were sampled, and 1551 valid responses were received, resulting in a response rate of 93.32%. In 2020, 804 permanent residents from a specific company in the 4th Division outside the Urumqi area were sampled and 765 valid responses were received, resulting in a response rate of 95.15%.

During the data collection process, the missing data were deleted and not included in the statistical analysis.

### 2.2. Survey methods

All healthcare professionals participating in the study underwent a series of assessments, including questionnaire surveys, physical examinations, and quality control training. The questionnaire encompassed various demographic data such as age, gender, ethnicity, education level, and marital status. Additionally, it included relevant information regarding the participants’ chronic disease history and diabetes-related details, including treatment medications and quality control measures. Physical examinations were conducted to assess participants’ height, weight, and blood pressure.

Blood-related tests were performed by using specific methods. Fasting plasma glucose levels were measured using the glucose oxidase method, and serum total cholesterol levels were assessed using the cholesterol oxidase method. High-density lipoprotein cholesterol (HDL-C) and low-density lipoprotein cholesterol (LDL-C) levels were measured directly using surfactant reagents. Triglyceride (TG) levels were determined using the glycerol phosphate oxidase method. Fasting blood samples were collected from the participants after a 12-hour fasting period. The collected whole blood samples were left at room temperature for 45 minutes before testing in a local laboratory at the brigade (or township) level. The testing instrument employed was 7600-020 fully automated analyzer manufactured by Hitachi, Ibaraki Prefecture, Japan.

Blood pressure measurements were obtained using an Omron electronic sphygmomanometer. Participants were instructed to rest for at least 5 minutes, and 3 measurements were taken with a 1-minute interval between each measurement. The average values of the three measurements were recorded.

The survey was conducted with the approval of the Medical Ethics Committee of our hospital.

### 2.3. Diagnostic criteria and relevant definitions

Body mass index (BMI) = body weight/(height × 2) (weight: kg, height: m). Smoking: Smoking ≥ 5 cigarettes per day. T2DM treatment: Use of any medication for T2DM treatment consistently within 2 weeks, including Chinese patent medicine and Chinese herbal medicine with hypoglycemic effects. Dyslipidemia^[6]^: TG ≥ 2.26 mmol/L indicates hypertriglyceridemia; total cholesterol ≥ 6.22 mmol/L indicates hypercholesterolemia; HDL-C < 1.04 mmol/L indicates low HDL-C; LDL-C ≥ 4.14 mmol/L indicates high LDL-C; individuals with any of the above conditions or diagnosed by a county-level or higher hospital and currently taking lipid-lowering drugs are considered to have dyslipidemia. Hypertension: patients with a confirmed diagnosis of hypertension in the past; hypertension diagnosis criteria,^[7]^ excluding secondary hypertension, severe organ diseases, arrhythmias, atrial fibrillation, second-degree or higher atrioventricular block, and severe liver or kidney dysfunction; and patients using any medication for hypertension treatment, including Chinese patent medicine and Chinese herbal medicine. The population with comorbid hypertension was classified into three categories: Category 1: patients without hypertension; Category 2: patients with comorbid hypertension but not receiving treatment; and Category 3: patients with comorbid hypertension and receiving treatment. Fasting Plasma Glucose target: Refers to a fasting period of at least 8 hours without calorie intake, and the blood glucose level measured using the glucose oxidase method falls between 4.4 and 7.0 mmol/L.

### 2.4. Statistical methods

This study used the SPSS software package (version 25.0) (International Business Machines Corporation [IBM], Armonk) for statistical analysis. The normality of continuous variables was assessed using the Kolmogorov–Smirnov (K-S) test, and it was determined that all continuous variables in this study followed a normal distribution (*P* > .05). Group comparisons were performed using *t* tests for continuous variables, whereas categorical variables were represented using frequencies and rates. Group comparisons for categorical variables were performed using the chi-square test. Multivariate analysis was performed using binary logistic regression to assess the independent risk factors influencing FBG achievement, with a significance level of *P* < .05 indicating statistical significance. Function plotting was conducted using Origin software.

## 3. Results

### 3.1. General information

This study enrolled a total of 2316 participants, with an average age of 66.72 ± 11.83 years. Of the participants, 1072 were male, accounting for 45.12% of the total sample. Of the total participants, 1418 (61.23%) were aged ≥ 65 years. The Han ethnicity comprised 2031 participants, representing 87.69% of the total sample. The population of the area surrounding Urumqi consisted of 1551 participants, accounting for 66.97%. At baseline, there were statistically significant differences (*P* < .05) between male and female patients with T2DM in terms of age, systolic blood pressure, diastolic blood pressure, ethnicity, region, education level, smoking status, marital status, and comorbid hypertension. For specific data, please refer to Table 1.

**Table 1:** General basic characteristics of the survey participants.

Variables	Male	Female	*t*/χ^2^	*P*
Age	65.78 ± 11.91	68.78 ± 10.12	−6.551	<.01
SBP (mm Hg)	132.87 ± 16.26	135.23 ± 16.23	−3.466	<.01
DBP (mm Hg)	78.6 ± 11.13	77.44 ± 10.76	2.545	.011
BMI (kg/m^2^)	25.83 ± 2.99	26.01 ± 3.63	−1.273	.203
Nationality			5.514	.019
Han	934 (89.46)	1097 (86.24)		
Minority	110 (10.54)	175 (13.76)		
Area			5.814	.016
Surrounding area of Urumqi	672 (64.37)	879 (69.10)		
Outside the Urumqi surrounding area	372 (35.63)	393 (30.90)		
Education			130.283	<.01
Below high school	375 (35.92)	760 (59.75)		
High school and above	669 (64.08)	512 (40.35)		
Smoke			397.799	<.01
No	725 (69.44)	1256 (98.74)		
Yes	319 (30.66)	16 (1.36)		
Marriage			95.370	<.01
Married	946 (90.61)	979 (76.97)		
Single	11 (1.05)	2 (0.16)		
Divorced or widowed	87 (8.33)	291 (22.88)		
Hypertension			12.979	<.01
No	278 (26.63)	258 (20.28)		
Yes	766 (73.37)	1014 (79.72)		

BMI = body mass index, DBP = diastolic blood pressure, SBP = systolic blood pressure.

### 3.2. Analysis of medication usage in T2DM patients

**Figure 1.**
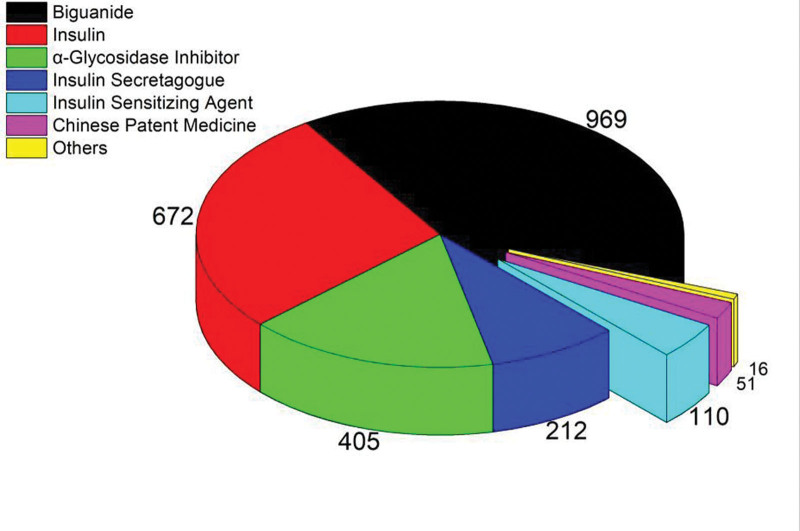
Number and proportion of drugs in T2DM patients. T2DM = type 2 diabetes mellitus.

Among the surveyed subjects, 1663 used medication for the treatment of T2DM. The main types of medications used were in the following order: metformin, insulin, and α-glucosidase inhibitors. Figure 1 shows the number of cases and the proportion of medication usage in T2DM patients.

### 3.3. Achievement of comprehensive control goals in T2DM

In this study, the treatment rate for T2DM patients was 71.80%. However, the rate of achieving the FBG target is the most important indicator of comprehensive control goals for T2DM. Among the surveyed subjects, 648 individuals achieved the FBG target, resulting in a rate of only 27.98%. When combining FBG levels with other indicators, the highest rate was the achieved with combined blood pressure (30.71%), while the lowest rate was the achieved with combined BMI (13.27%). For more specific data, please refer to Table 2.

**Table 2:** The comprehensive control compliance of FBG combined with other targets.

Key indicators	Joint indicators	Standard	Case number	Control rate (%)
FBG	HDL-C	>1 mmol/L, >1.3 mmol/L*	131	20.22
	LDL-C	<2.6 mmol/L, 1.8 mmol/L†	125	19.29
	TG	<1.7 mmol/L	130	20.06
	TC	<4.5 mmol/L	111	17.13
	BMI	<24.0 kg/m^2^	86	13.27
	Blood pressure	<130/80 mm Hg	199	30.71

BMI = body mass index, FBG = fasting blood glucose, HDL-C = high-density lipoprotein cholesterol, LDL-C = low-density lipoprotein cholesterol, T2DM = type 2 diabetes mellitus, TC = total cholesterol, TG = triglyceride.

* Male > 1 mmol/L, female > 1.3 mmol/L.

† No atherosclerotic heart disease < 2.6 mmol/L, with atherosclerotic heart disease < 1.8 mmol/L.

### 3.4. Single-factor analysis of blood glucose achievement in T2DM patients

Using the achievement of the FBG target as the dependent variable and the different characteristics of T2DM populations as risk factors, a single-factor analysis was conducted. The results showed that there were statistically significant differences (*P* < .05) in BMI, ethnicity, region, presence of hypertension, presence of dyslipidemia, and use of glucose-lowering therapy among the different groups. The higher the BMI, the lower was the FBG achievement rate. The FBG achievement rate was lower in the minority ethnic groups than in the Han ethnic group. T2DM patients from areas outside Urumqi had a lower FBG achievement rate than those from areas surrounding Urumqi. Patients without hypertension had a higher FBG achievement rate, whereas those with hypertension, but without treatment, had a lower FBG achievement rate. Patients with dyslipidemia had a lower FBG achievement rate than those with normal lipid levels. Patients who did not receive treatment for diabetes had a lower blood glucose achievement rate than treated patients. Please refer to Table 3 for further details.

**Table 3:** Single factor analysis of FBG control rates in patients.

Variables	Total number	Control number	Control rate (%)	*t*/χ^2^	*P*
Age				0.857	.354
<65	898	261	29.1		
≥65	1418	387	27.3		
Gender				1.268	.260
Male	1044	280	26.8		
Female	1272	368	28.9		
BMI (kg/m^2^)				17.316	<.01
<24	651	218	33.5		
24–27.99	1125	307	27.3		
≥28	540	123	22.8		
Nationality				8.542	<.01
Han	2031	589	29.0		
Minority	285	59	20.7		
Area				15.531	<.01
Surrounding area of Urumqi	1551	474	30.6		
Outside the Urumqi surrounding area	765	174	22.7		
Education				0.957	.328
Below high school	1135	307	27.0		
High school and above	1181	341	28.9		
Smoke				1.035	.309
No	1981	562	28.4		
Yes	335	86	25.7		
Marriage				2.804	.246
Married	1925	549	28.5		
Single	13	5	38.5		
Divorced or widowed	378	94	24.9		
Hypertension				6.164	.046
Type 1	536	163	30.4		
Type 2	471	115	24.4		
Type 3	1309	370	28.3		
Dyslipidemia				22.419	<.01
No	370	141	38.1		
Yes	1946	507	26.1		
Hyperglycemia therapy				10.639	<.01
No	653	151	23.1		
Yes	1663	497	29.9		

BMI = body mass index, FBG = fasting blood glucose.

### 3.5. Multifactor analysis of factors affecting blood glucose control in T2DM patients

The FBG level that reached the target value was used as a dependent variable. Considering professional knowledge and conducting univariate analysis, factors such as age, geographical location, BMI, ethnicity, T2DM treatment status, and hypertension were included as independent variables in a logistic regression model for multifactor analysis. The results showed that living outside of Urumqi, older age, higher BMI, abnormal blood lipids, and untreated hypertension were independent risk factors affecting blood glucose control (OR < 1), and these factors were statistically significant (*P* < .05). There was no statistically significant difference (*P* > .05) in FBG levels between the patients with and without hypertension. T2DM treatment was a protective factor affecting blood glucose control (OR > 1) and this factor was statistically significant (*P* < .05). Please refer to Table 4 for further details.

**Table 4:** Multivariate logistic regression analysis of influencing FBG control rates in patients.

Variables		β	SE	Wald χ^2^	*P*	OR	OR 95% CI	
Area	Surrounding area of Urumqi					1.000		
	outside the Urumqi surrounding area	−0.397	0.108	13.434	<.01	0.673	0.544	0.831
Age	<65					1.000		
	≥65	−0.243	0.100	5.887	.015	0.784	0.644	0.954
BMI	<24					1.000		
	24–27.99	−0.262	0.108	5.908	.015	0.770	0.623	0.951
	≥28	−0.453	0.136	11.096	<.01	0.636	0.487	0.83
Nationality	Han					1.000		
	Minority	−0.243	0.162	2.261	.133	0.784	0.571	1.077
Hyperglycemia therapy	No					1.000		
	Yes	0.445	0.121	13.630	<.01	1.561	1.232	1.977
Dyslipidemia	No							
	Yes	−0.448	0.122	13.548	<.01	0.639	0.503	0.811
Hypertension	Type 1					1.000		
	Type 2	−0.334	0.143	5.469	.019	0.716	0.541	0.947
	Type 3	0.089	0.124	.509	.476	1.093	0.856	1.395
Constant		−0.312	0.222	1.978	.160	0.732		

BMI = body mass index, FBG = fasting blood glucose.

## 4. Conclusions

The treatment and fasting blood glucose control rates of T2DM patients in the Xinjiang Production and Construction Corps were relatively low. Living outside Urumqi, age 65 years or older, BMI ≥ 24, abnormal blood lipid levels, and untreated hypertension were independent risk factors affecting fasting blood glucose control in patients. Medication is a protective factor that affects blood glucose levels. The main medications used in treatment include metformin, insulin, and alpha-glucosidase inhibitors.

## 5. Discussion

This study was a multi-stage cluster sampling survey conducted on 2316 T2DM patients in the Xinjiang Production and Construction Corps from 2019 to 2020. Therefore, the study results can demonstrate the medication, blood glucose control, and risk factors of T2DM patients in the Xinjiang Corps region. The study analyzed the T2DM medications used by 1663 individuals, with the highest number of cases using metformin (metformin tablets/extended-release metformin tablets), followed by insulin and the third-ranked α-glucosidase inhibitors (acarbose tablets), which is consistent with the recommended medications in guidelines and literature reports.^[8–10]^ However, during the survey, it was found that there were still many irrational medication practices. In addition to common issues such as improper dosage, other prominent problems included 16 cases of patients using non-drugs for T2DM treatment, with their FBG not meeting the target, and 6 cases of patients using both repaglinide and Xiaoke Pill, which are considered duplicate medications. Therefore, patient education on medication compliance is important.

The study found that the treatment rate of T2DM patients in the Corps was 71.80%, and the FBG target rate was 27.98%. The highest reported T2DM treatment rate in the literature was 95.0% among some residents of Fujian Province in 2017.^[11]^ The treatment rates in economically developed provinces and cities such as Chongqing^[12]^ and Zhejiang^[13]^ were generally between 60% and 80%. The treatment rate reported in a 2017 study of 4608 people in Xinjiang was 21.31%.^[14]^ In terms of FBG target rate, the blood glucose target rate among people aged 40 and above in Jilin Province was as high as 75.9%,^[15]^ followed by a target rate of 57.6% in Jiangxi Province in 2014,^[16]^ and the FBG target rate among the Uygur residents of Xinjiang was only 5.57%.^[14]^ The treatment rate of T2DM patients in the Corps was slightly higher than that in other provinces, but the target rate is lower. Possible reasons for this include: This study defined the use of any Western medicine, traditional Chinese medicine, or herbal medicine for T2DM treatment within two weeks as receiving treatment, resulting in a higher treatment rate; The improvement of the national medical security system has benefited the Corps employees. In 2016, the Corps launched a comprehensive health examination project, which covered free medical examinations for all residents in the Corps area, thus achieving early detection and treatment; and The awareness of health care among Corps employees has increased, but there is still room for improvement in health management. The survey found that most patients only knew that T2DM requires medication, but due to factors such as medical conditions and economic conditions, they rarely monitor their blood glucose levels. Therefore, the treatment rate is high, but the target rate is low.

Primary care doctors should not only strive to improve the blood glucose management target rate of T2DM patients but also emphasize the comprehensive control of various risk factors such as blood pressure, blood lipids, and proteinuria, aiming for combined target achievement. Therefore, this study also conducted a combined analysis of other indicators of the FBG target achievement. The combined blood pressure target achievement rate was the highest, while the combined BMI target achievement rate was the lowest. At the same time, attention should be paid to LDL, which is an independent risk factor for cardiovascular and cerebrovascular diseases.^[17,18]^ The target achievement rate was only 19.29%. This indicates that LDL, hypertension, and BMI target achievement are important indicators when formulating intervention measures for patients.

In China, 30% to 45% of hypertensive patients also have diabetes,^[19]^ and the prevalence of hypertension increases with abnormal blood lipids.^[20,21]^ Diabetes or abnormal blood lipids can cause changes in vascular constriction function and blood viscosity, thereby inducing or exacerbating hypertension.^[22,23]^ Univariate analysis found that patients without hypertension had a higher FBG target achievement rate, whereas patients with hypertension but without treatment had a lower FBG target achievement rate. In contrast, patients with abnormal blood lipid levels had a lower FBG target achievement rate than those with normal blood lipid levels, indicating that other diseases have a significant impact on the FBG target achievement rate. Once diabetes patients develop other diseases, achieving an FBG target becomes more difficult.^[24]^ Among other factors that were statistically significant, high BMI, ethnic minorities, and T2DM patients outside the Urumqi area had lower FBG target achievement rates, which may be related to economic development, religious beliefs, language and culture, lifestyle habits, and genetic background.^[25–27]^ Multivariate analysis found that being outside the Urumqi area, older age, high BMI, abnormal blood lipids, and untreated hypertension were independent risk factors affecting blood glucose target achievement, whereas T2DM treatment was a protective factor.^[28,29]^ The relative shortage of medical resources outside Urumqi may lead to increased difficulties for patients receiving treatment and managing diseases, as patients may not have access to timely medical services and medications, thus affecting their ability to control blood sugar levels, Contrary to foreign research findings,^[30,31]^ this study suggests that older patients have poorer blood glucose control, possibly due to the fact that patients aged 65 and above in this region were born before 1955, have lower educational levels, and exhibit poor compliance with treatment, which is consistent with an analysis of diabetes self-efficacy management among residents of three provinces in China.^[32]^ Several studies have indicated^[33,34]^ that common blood lipid abnormalities in patients with T2DM mainly include abnormal levels of TG and HDL-C; elevated TG and low HDL-C levels can lead to insulin resistance, resulting in poor blood glucose control and forming a vicious cycle. A meta-analysis in 2023 suggested^[35]^ that the most widely used lipid-lowering statin drugs slightly but significantly increase blood glucose levels and affect blood glucose control; although the diabetogenic effect of statins is not fully understood, some potential mechanisms have been proposed, such as their possible association with elevated blood glucose concentrations, decreased insulin secretion, and insulin resistance. Nevertheless, guidelines from the United States and other international sources^[36,37]^ still recommend the use of statin drugs for the treatment of all adult diabetic patients; this indicates that, overall, the benefits of using statins to treat lipid abnormalities in diabetic patients outweigh the risks. However, this study had a small number of cases for the treatment of lipid abnormalities, so a thorough investigation into the impact of lipid abnormality treatment on blood glucose control was not conducted. With hypertension or dyslipidemia in patients can elevate the risk of cardiovascular diseases, further complicating blood sugar control for diabetic patients as well as patients’ own conditions and disease treatment, patient health management is also crucial. This suggests that when formulating intervention measures, we should focus on health education, inform patients about the connection and impact of various indicators, including diseases, and raise patients’ awareness of health management, especially in controlling weight and improving medication compliance.^[38,39]^

The battlefield for the prevention and treatment of chronic diseases is at the primary level. Although the chronic disease community management system in the Corps has achieved some results, it still lags far behind that of the developed regions. Exploring how to make the best use of limited medical resources, maximizing the functions of community and grassroots medical workers such as team doctors, strengthening publicity and education, improving unhealthy habits, enhancing patients’ health awareness and compliance, and changing the above-mentioned risk factors through health education and standardized management control will be the key issues to be addressed by our research group in the next step.^[40]^

The article also has certain limitations. One limitation is that glycated hemoglobin is the gold standard for monitoring blood sugar control, but this article chose fasting blood glucose as the data indicator for blood sugar control. This is because the detection of hemoglobin should use standardized methods and strict quality control; if not met, there may be a higher false positive rate. Additionally, fasting blood glucose is closely related to glycated hemoglobin and has reasonable sensitivity and specificity for detecting poor blood sugar control; fasting blood glucose remains the primary blood sugar monitoring indicator for low-income diabetes patients in the current era.^[41,42]^ Another limitation is that the cross-sectional design can only suggest that hypertension and blood lipid abnormalities are risk factors for blood sugar control, and cannot evaluate their exact causal relationship. However, this study also has certain strengths. This series of surveys was conducted among a large population in the underdeveloped Xinjiang Production and Construction Corps region of China, which includes a wide geographical area and diverse ethnic groups, with relatively uniform demographic characteristics among the study population. In summary, owing to the wide geographical distribution, scattered population, and uneven distribution of medical resources in Xinjiang, the treatment and target achievement rates of T2DM patients still need to be further improved.^[43]^ Our research group will monitor the progress and risk factors of T2DM prevention and treatment work at the grassroots level in the Corps, promote the translation of the core diagnosis and treatment contents of diabetes prevention and treatment guidelines into clinical practice, and implement them in the majority of patients with diabetes. We plan to conduct targeted interventions in various grassroots medical institutions for populations affected by the above-mentioned risk factors affecting FBG control. Simultaneously, we actively explored new appropriate prevention and treatment models and methods for T2DM.

## Author contributions

**Conceptualization:** Hui Li, Na Tang.

**Data curation:** Ye Hong, Na Tang.

**Formal analysis:** Ye Hong, Jie-ping Chen.

**Funding acquisition:** Hui Li, Nan-yue Kuang.

**Investigation:** Jie-ping Chen.

**Methodology:** Nan-yue Kuang, Hui Li.

**Project administration:** Hui Li.

**Resources:** Na Tang.

**Writing – original draft:** Nan-yue Kuang.

**Writing – review & editing:** Nan-yue Kuang.

## Correction

The first author’s name was incorrectly spelled as Nang-yue Kuang. It has been corrected to Nan-yue Kuang in the byline and the author contributions.
